# Pharmacokinetic model selection for infliximab based on inflammatory bowel disease phenotype and severity: Toward model-informed precision dosing

**DOI:** 10.1371/journal.pone.0352967

**Published:** 2026-07-24

**Authors:** Ana Homšek Ilić, Marija Jovanović, Srđan Marković, Sandra Vezmar Kovačević, Tamara Knežević Ivanovski, Đorđe Kralj, Petar Svorcan, Katarina Vučićević

**Affiliations:** 1 Department of Pharmacokinetics and Clinical Pharmacy, University of Belgrade - Faculty of Pharmacy, Belgrade, Republic of Serbia; 2 University Hospital Medical Centre “Zvezdara”, Department of Gastroenterology and Hepatology, Belgrade, Republic of Serbia; 3 University of Belgrade - Faculty of Medicine, Belgrade, Republic of Serbia; PLOS ONE, UNITED KINGDOM OF GREAT BRITAIN AND NORTHERN IRELAND

## Abstract

Ulcerative colitis (UC) and Crohn’s disease (CD) differ in intestinal location and depth of involvement. Patients with UC who have or experienced a flare-up according to Truelove–Witts criteria (ACUS), as well as those with fistulizing Crohn’s disease (FIST), may represent difficult-to-treat populations. This study investigates the phenotype- and severity-driven selection of infliximab (IFX) population pharmacokinetic models to optimize predictive performance and support precision dosing in inflammatory bowel disease (IBD) care. A retrospective analysis was conducted at University Hospital Medical Centre “Zvezdara”. Twenty published IFX models were evaluated across five datasets (overall, ASUC, CD, FIST, UC), primarily using trough concentrations obtained via routine therapeutic drug monitoring. Predictive accuracy was assessed using median prediction error (MDPE) and median absolute prediction error (MAPE). Bayesian forecasts were evaluated using median individual prediction error (MDIPE) and its absolute value (MAIPE). Normalized prediction distribution errors (NPDE) and visual predictive check (VPC) were used for simulation-based diagnostics. Moreover, probability of probability of target-attainment (PTA%) per model and subgroup was calculated. No model met all predefined prediction-based performance criteria. In *a priori* analysis, the Matsuoka model showed the satisfactory overall performance, while the Xu model was most suitable for CD patients. In *a posteriori* analysis, the Ternant 2008 model had the well overall accuracy, with other models (Dreesen 2021 for ASUC, Matsuoka for CD, Brandse 2016 for UC) performing satisfactory in respective subgroups. Simulation NPDE diagnostics identified the Aubourg model as suitable for the overall dataset, while VPC results mainly supported the *a priori* analysis conclusions. These findings highlight the relevance of disease phenotype and severity in selecting IFX pharmacokinetic models. Rather than relying on a one-size-fits-all approach, tailoring model choice to patient subgroups enhances predictive performance. This supports a more individualized, model-informed precision dosing (MIPD) strategy for optimizing IFX dosing in IBD.

## Introduction

Inflammatory bowel disease (IBD) is characterized by a chronic inflammation of the intestines, which arises from an atypical immune response to the gut microbiota. IBD is primarily categorized into two distinct types based on the location of the disease and the extent of tissue involvement in the intestinal wall. Typically followed by an unpredictable relapsing-remitting course, significantly impairing patients’ quality of life and placing a considerable burden on both the healthcare system and workplace productivity [1,2].

Ulcerative colitis (UC) is an idiopathic inflammatory disorder of the colon that develops as a consequence of diffuse friability and surface erosions on the wall accompanied by bleeding. The inflammation is limited to the mucosa and submucosa, usually starting in the rectum and extending proximally continuously throughout the colon [3]. In contrast, Crohn’s disease (CD) can develop in any part of the gut from the mouth to the anus. CD can be located on the ileocecal portion, colon, terminal ileum, small bowel and anorectal part, rarely gastroduodenal or oral, involving one or multiple areas, with “skip lesions” in between which remain unaffected. The affected areas become thickened and narrow, involving the entire thickness of the gut wall, progressing to deep fissuring ulcers, fibrosis and strictures, followed by abscesses and fistulae [2,4].

Each type of IBD can be classified by disease severity as mild, moderate, or severe [1]. Acute severe ulcerative colitis (ASUC) is defined as a disease flare up which presents itself as six or more bloody stools per day, accompanied by at least one sign of systemic toxicity – such as fever, haemoglobin level below 105 g/L, erythrocyte sedimentation rate exceeding 30 mm/h, or a heart rate over 90 bpm [5]. Approximately one-third of patients who experienced ASUC fail to achieve remission with corticosteroid therapy, in which case infliximab (IFX) is one of recommended rescue treatments. Nevertheless, a subset of patients remains unresponsive [6–8]. Fistulizing Crohn’s disease (FIST) is observed in one-third of CD patients after formation of granulation tissue tract between two epithelial lined surfaces [9,10]. These patients often experience more complex and burdensome symptoms compared to those with non-fistulizing CD. Given the presence of elevated levels of tumor necrosis factor (TNF) around the fistula, IFX has become a mainstay in fistula management and remains one of the most effective therapeutic options [10,11]. However, treatment failure and recurrence of fistulas remain common, often necessitating surgical interventions. We hypothesized that patients with prior UC who experienced a flare-up and patients with fistulizing CD represent difficult-to-treat and clinically distinct subgroups, with potentially different IFX exposure even after resolution of the acute condition, compared to general UC and CD populations, and that different models may be more appropriate for each.

Despite tremendous efforts to stabilize patients with specific forms of IBD, clinicians continue to face considerable challenges in maintaining long-term symptom control, even with the support of therapeutic drug monitoring (TDM). To manage each patient individually, model informed precision dosing (MIPD), which integrates TDM with developed pharmacometric models, has been introduced to support dose and regimen individualization. Over nearly three decades since IFX has been approved [12], numerous population pharmacokinetic (PK) models have been developed to describe its disposition and variability among patients. Moreover, several studies have evaluated the applicability of these models in real-world clinical settings [13–16]. However, to date, limited studies have systematically compared the predictive performance of IFX model across patient subgroups defined by different disease modalities or severity.

Given the substantial number of patients presenting with these complex forms, and the distinct PK behaviour of IFX across different IBD modalities, we aim to assess the suitability of existing IFX models for use in a real-world clinical setting. Specifically, the study sought to assess the models’ capacity for *a priori* and *a posteriori* prediction in overall and various subgroups of patients, as well as their utility in simulation-based application.

## Methods

### Model selection

Review of the available literature was conducted using PubMed database to gather all papers describing PK of IFX. Only publications in English were considered. The following query used for the search was last performed in April 2025: (Infliximab) AND (pharmacokinetic model OR population pharmacokinetics OR population pharmacokinetic model* OR nonlinear mixed effects model* OR NONMEM OR pharmacometric) AND (Inflammatory Bowel Diseases OR Crohn Disease OR Crohn’s Disease OR ulcerative colitis).

In addition, a manual search for relevant papers was performed by checking reference lists of some articles. Exclusion criteria for papers were:

1) Papers not based on nonlinear mixed effects modelling of IFX in IBD,
2) Papers based on non-representative populations (paediatric, other indications, etc.),
3) Papers that used previously developed models,
4) Review or methodological papers, conference abstracts.

Information on the structure of the PK model, PK parameter values, significant covariate effects, inter-individual variability, inter-occasion variability and residual variability was obtained from each publication. If the publication had provided NONMEM codes in the supplementary material, these were adapted and implemented on the evaluation dataset.

### Evaluation datasets

Data were retrospectively extracted from the medical charts of patients at the Department of Gastroenterology and Hepatology of the University Hospital Medical Centre “Zvezdara” in Belgrade, focusing on individuals treated with IFX. The design and conduct of this research were in accordance with the ethical standards outlined in the Declaration of Helsinki. The research protocol was approved by the institutional Ethics Committee (No. IRB00009457, in October 5, 2022). All necessary information was extracted from the patients’ medical records, including: IFX dosing information from the first administered dose, blood sampling information for TDM with exact dates and measured IFX concentration, patient characteristics (concomitant immunomodulatory drugs, prior administration of biologics, clinical and laboratory assessments). From medical records complete blood cell count, inflammation marker (in particular faecal calprotectin – FCP, CRP) were available. IFX concentrations available at the IBD Centre were evaluated. Patients treated by IFX and diagnosed with CD with or without fistulas (observed at physical examination), as well as patients with UC including those who presented at some point with an acute severe relapse (as determined by Trulove Witts criteria [5]) were included in the analysis. We divided all IBD patients into four subtypes: UC without or with current/prior ASUC (UC and ASUC), CD without or with perianal fistula (CD and FIST).

TDM was carried out at different timepoints during the IFX treatment. For most patients, the concentration was measured at week 14 and usually measurements were obtained at scheduled controls or in relapse (combination of proactive and reactive TDM). All concentrations were obtained right before the next IFX dose (trough concentrations). When measured concentration was low, a measurement of anti-drug antibodies (ADA) was performed. These measurements were performed using commercially available R-Biopharm^®^ ELISA tests on a Dynex DS2 analyzer (Dynex Technologies, USA) in the biochemical laboratory of the University Hospital Medical Centre “Zvezdara”.

Dosing of IFX varies according to the stage of treatment. In the beginning, at induction phase, the usual dosing regimen is at week 0, 2 and 6, but there is also an accelerated induction protocol where patients get doses every or every other week until week 14. Once induction is complete, in the maintenance phase IFX is administered every 4, 6 or 8 weeks, but patients with severe forms of disease can even get the drug every other week. Dose depends on the body weight of the patient starting from 5 mg/kg up to 10 mg/kg if necessary [17].

### Evaluation methods

**Fig 1:**
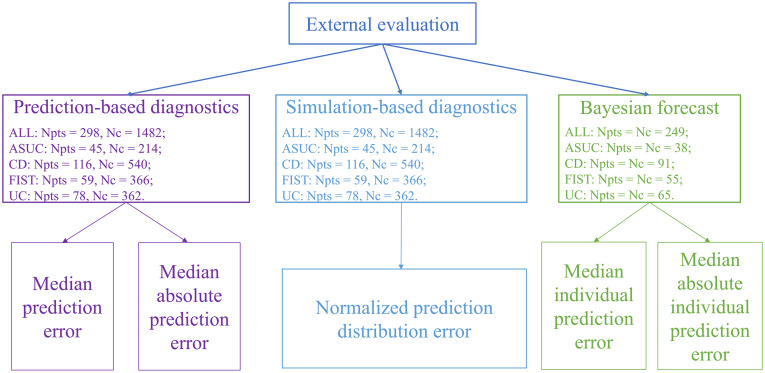
Evaluation strategy for predictive performance of infliximab (IFX) population pharmacokinetic (PK) models and used datasets. ALL – overall patients, ASUC – acute severe ulcerative colitis, CD – Crohn’s disease, FIST – fistulising Crohn’s disease, UC – ulcerative colitis; Npts – number of patients, Nc – number of concentrations.

All selected population PK models were separately implemented in NONMEM version 7.5 (ICON Development Solutions, MD, USA) for external evaluation. Each NONMEM code was adapted compiling equations and parameter values into the control file and executing with an iteration zero, by setting MAXEVAL to 0 in the code. All assessments of model performance were stratified based on the subpopulation to which patients were assigned. The R (ver. 4.2.2) with RStudio (ver. 2024.04.2 + 764 “Chocolate Cosmos”) program was used for pre- and post-processing of inputs/outputs. Missing data of some covariates were replaced in the code with the reference values from the model in question. As endoscopic evaluation data were not available within the validation dataset, variables based on endoscopic scoring systems were not considered. The methodological approaches of the evaluation, along with data used in each one, are presented in Fig 1.

### Prediction-based diagnostics

Prediction-based validation of a model was based on the comparison between predictions obtained from the model and IFX concentrations in the validation dataset. The predictions represent *a priori* predictions based on the patients’ covariates identified as relevant in each model. Several different metrics can be used for model evaluation [18–20]. The prediction error (PE) in each timepoint was obtained by Equation 1.


$$\begin{array}{c} {PE}_{i,j}=\frac{C_{i,j}^{pred}-C_{i,j}^{obs}}{C_{i,j}^{obs}} \end{array}$$ (1)

where *c*^*pred*^ is predicted*, c*^*obs*^ observed IFX concentration for each *i*^*th*^ individual and *j*^*th*^ measurement.

The bias and precision of predictions were evaluated using the median prediction error (MDPE, Equation 2) and the median absolute prediction error (MAPE, Equation 3), respectively.


$$\begin{array}{c} MDPE\;(\%)=\left\{median\left({PE}_{\text{1},\text{1}},\ldots \;,{PE}_{i,j}\right)\right\}\cdot \text{1}\text{0}\text{0}\% \end{array}$$ (2)

$$\begin{array}{c} MAPE\;(\%)=\left\{median\left(\left|{PE}_{\text{1},\text{1}}\right|,\ldots \;,\left|{PE}_{i,j}\right|\right)\right\}\cdot \text{1}\text{0}\text{0}\% \end{array}$$ (3)

Thresholds for predictive performance metrics were selected based on commonly applied criteria in the literature, as no universally standardized or evidence-based cut-offs exist. Specifically, values of ±20% for MDPE and <30% for MAPE were applied by some authors to indicate the accuracy and precision of the models [19–24]. However, slightly less stringent threshold (e.g., < 35%) for MAPE have also been used in external evaluation studies [25,26]. For each subpopulation this method was used to evaluate all identified models.

### Bayesian forecast analysis

Bayesian forecast with maximum *a posteriori* estimation was implemented to further assess predictive capabilities of models. Typical values of PK parameters, as well as inter-individual and residual variability, were set to the reported final values given in the respective paper. Individual PK parameters were calculated based on all prior to last measurements for all patients using first-order estimation with the POSTHOC option specified in the code. All previous concentration measurements were used to predict the final observed IFX concentration, as previous studies showed that prediction performance improves with the inclusion of multiple prior observations [27]. Individual predicted concentrations were calculated for the last timepoint of each patient and compared to the corresponding observed concentration to derive the individual prediction error (IPE). Patients with only a single measured concentration were excluded from this analysis, as the absence of prior observations precluded meaningful model-based prediction.

The bias and precision of prediction were evaluated using the median individual prediction error (MDIPE) and median absolute individual prediction error (MAIPE) respectively. Due to a lack of defined criteria for Bayesian forecast analysis in the literature, the same thresholds which were used for prediction-based analysis were applied to assess model accuracy and precision. For each subpopulation, this method was employed to evaluate the predictive performance of all selected models. In order to obtain clinically relevant results, probability of target attainment (PTA%) was calculated. Trough concentration target varies depending on the subpopulation, therefore, in CD and UC subgroups as well as the overall dataset, the target of above 7 μg/mL was implemented since it is the usual target for patients in maintenance treatment of IBD. For ASUC and FIST subcohort however, a target over 10 μg/mL was used since this criterion is reported in previous article [28] and is used on the clinic.

### Simulation-based diagnostics

The normalized prediction distribution error (NPDE) is the uncorrelated and normalized version of the prediction discrepancy and can be calculated using the inverse function of the normal cumulative density function [29]. In our research, NPDE was computed using the NPDE R package ver. 3.5 (available on: https://CRAN.R-project.org/package=npde). Simulated data files were obtained in NONMEM by implementing 1000 simulations with the fixed final parameters of the chosen models. In order to identify a model as adequate, NPDE should follow a standard normal distribution with a mean of 0 and a deviation of 1. The hypothesis of a normal distribution was verified or rejected based on Students’ t-test for means, Fisher’s variance test and the Shapiro-Wilks test for normality. Additionally, visual predicative checks (VPC) have been generated for each model in every subgroup using 200 samples and automatic binning, and plotted against time since last dose.

## Results

### Selected population pharmacokinetic models

**Fig 2:**
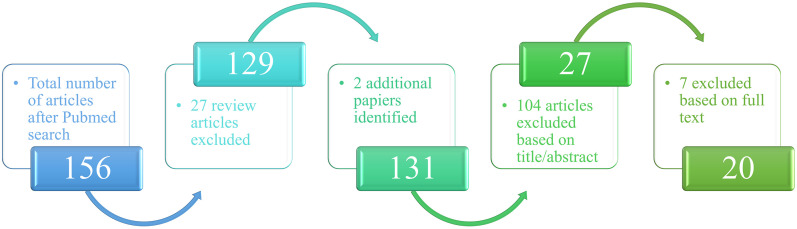
Flowchart of the articles screening and infliximab (IFX) pharmacokinetics model selection.

Among 156 papers published between 2003 and 2025, twenty population PK models were collected using the PubMed database (Fig 2). Most of the selected models were originally developed using NONMEM; however, five models were developed with Monolix (Lixoft), and one using WinNonmix (Pharsight). Nevertheless, all models were adapted and implemented in NONMEM for application to our dataset.

All selected publications were published in the last two decades, which coincides with the approval of IFX for IBD. The chosen models included data from either real-world settings or randomised clinical trials (Table 1). Real-world data were collected retrospectively or prospectively; however, two studies [30,31] did not specify the nature of data collection. Five studies were conducted across multiple hospital centres, while the majority (ten studies) were performed in a single hospital. Among the five publications utilizing clinical trial data, all but one [32] were multicentre studies. Each publication included IBD population, either solely or as a majority, as required by the search criteria. Nevertheless, some studies focused exclusively on either UC or CD patients. Additionally, two publications [33,34] incorporated patients with rheumatoid arthritis, ankylosing spondylitis, psoriatic arthritis and Kawasaki disease alongside IBD patients.

**Table 1:** Selected population pharmacokinetic (PK) infliximab (IFX) models for the evaluation [30–49].

No.	First author ^a^	Study type (No. of centres)	Diagnosis	Pharmacokinetic model data					
				Software	COMP	CL; Q [L/day] (BSV %)	Vc; Vp [L] (BSV %)	Covariates on CL	Covariates on Vc
	Aubourg	Retrospective (1)	CD, UC	MNL	2	F: 0.336 M: 0.456 (47); 1.992	F: 2.6 M: 3.2 (27); 4.5	sex	sex, BW
2.	Brandse 2016	Prospective (2)	UC	NM	2	0.54 (24); 0.51	3.3 (20); 2.2 (42)	ADA + , ALB	/
3.	Brandse 2017	Retrospective (1)	CD, UC	NM	2	0.359 (38.1); 0.0697	4.72 (68.6); 2.4 (71,7)	BW, ALB, ADA + , ATNF	BW
4.	Buurman	Retrospective (1)	CD, UC	NM	2	0.199 (18); 0.0618	4.94 (17.1); 3.13	ADA + , sex, PHASE	HBI
5.	Dotan	Prospective (1)	CD, UC	NM	2	0.381 (13.45); 0.122	2.37 (42.07); 1.37 (32.4)	BW, ALB, ADA+	BW
6.	Dreesen 2019	Retrospective (multi)	UC	NM	1	0.353^#^ (29.2)	6.34 (26)	CRP, Mayo, ALB	FFM, PAN, CS
7.	Dreesen 2021	RCT (multi)	CD	NM	2	0.277 (28.5); 0.0201	4.90; 0.844	ADA + , FCP, ALB, CDAI	/
8.	Edlund	RCT (multi)	CD	NM	2	0.298 (33.3); 0.147	3.56 (12.6); 1.27 (55.3)	BW, ADA+	BW
9.	Fasanmade 2009	RCT (multi)	UC	NM	2	0.407 (37.68); 7.14	3.29 (22.11); 4.13	ALB, ADA + , sex	sex, BW
10.	Fasanmade 2011	RCT (multi)	CD	NM	2	0.369^*^ (30.7); 0.154^*^	3.58^*^ (12.6); 1.33^*^ (55.3)	ALB, ADA + , IMD, BW	BW
11.	Grisic	RCT (1)	CD, UC	NM	2	0.262 (34.9); 0.161	3.67 (12.8); 0.956 (55.3)	ADA + , ALB, BW, IMD	/
12.	Kantasiripitak	Retrospective (1)	CD, UC	NM	2	0.278 (29.6); 0.0597	5.03 (81.5); 3.04	age, FFM, ALB, CRP, ADA+	/
13.	Magro	Prospective (multi)	CD, UC	NM	2	0.275 (18.2); 0.158	3.67 (40); 1.37 (50)	BW, ALB, ADA + , FCP	/
14.	Matsuoka	Real-world (1)	CD	NM	1	0.432 (33.2)	7.35 (40)	BW, ADA + , ALB	/
15.	Passot	Retrospective (1)	CD, UC	MNL	1	0.23 (30.4)	5.2 (22.4)	sex, BW, IBD	sex, BW, IBD
16.	Petitcollin	Real-world (1)	CD, UC	MNL	1	0.273 (44.3)	11.5 (25.4)	CRP, IBD, D, AZA, PMS	/
17.	Ternant 2008	Retrospective (1)	CD, UC	WNM	2	ADA-: 0.288 (27.4) ADA + : 0.768 (22.5); 0.1296 (10)	F: 1.1 (11.3) M: 2.3 (14.1); 1.9 (15)	ADA+	sex, BW
18.	Ternant 2015	Prospective (multi)	CD	MNL	1	0.2989^c^	6.1^b^	hsCRP, FCGR3A	/
19.	Ternant 2018	Retrospective (1)	CD, UC, RA, PsA, As	MNL	1	0.24 (32)	5.3 (20)	BW, IBD, RA, MTX	sex, IBD
20.	Xu	Prospective (multi)	CD, UC, RA Kw	NM	2	0.294 (32.7); 0.0719 (110)	3.33 (15); 1.14 (79.9)	BW, ADA + , ALB	BW

ADA- – anti-drug antibody negative patients; ADA + – anti-drug antibody positive patients; ALB – albumin level; As – ankylosing spondylitis; ATNF – previous treatment with anti-TNF monoclonal antibodies; AZA – concomitant administration of azathioprine; BSV – between-subject variability; BW – body weight; CD – Crohn’s disease; CDAI – Crohn’s disease activity index score; CL – clearance; COMP – number of compartments; CRP – level of C-reactive protein; CS – concomitant administration of corticosteroids; D – dose; F – female; FCGR3A – gene encoding low affinity immunoglobulin gamma Fc region receptor III-A; FCP – faecal calprotectin level; FFM – fat free mass; HBI – Harvey-Bradshaw index; hsCRP – high sensitivity CRP; IBD – type of inflammatory bowel disease; IMD – concomitant administration of immunomodulatory drugs; Kw – Kawasaki disease; M – male; Mayo – Mayo subscore; MNL – Monolix; MTX – concomitant methotrexate administration; NM – NONMEM; No. – number; PAN – patients with pancolitis; PHASE – induction or maintenance phase of treatment; PMS – partial Mayo score; PsA – psoriatic arthritis; Q – intercompartmental clearance; RA – rheumatoid arthritis; RCT – randomized controlled trial; UC – Ulcerative colitis; Vc – central volume of distribution; Vp – peripheral volume of distribution; WNM – WinNonmix.

^a^Year of the publication is indicated if multiple publications of the same first author are present.

^b^Calculated for a patient with median body weight.

^c^Calculated from typical value of ke and Vc.

Among the 20 selected papers, the majority (14) described IFX disposition with two-compartment models, while six employed one-compartment model (Table 1). Most of the one-compartment models were developed using only trough concentrations of IFX, except for one study [35] that also included mid-interval concentrations. Conversely, one single two-compartment model was based exclusively on trough concentrations [36]; all others, in addition to trough, included either peak or mid-interval concentrations as well.

Based on selected models, typical IFX clearance values ranged from 0.199 to 0.54 L/day [36,38], but intercompartmental clearance (where applicable) was the most variable parameter varying from 0.0201 to 7.14 L/day [41,43]. Central volume of distribution in two-compartment models was estimated to be between 1.1 and 5.03 L [45,48], and, innately, slightly higher in one-compartment models (5.2–11.5 L) [31,47]. Volume of distribution of the peripheral compartment varied considerably (0.844–4.13 L) [41,43]. Between-subject variability is presented in Table 1. Inter-occasion variability was accounted for in two articles, Dreesen et al. [49] with an effect of the dosing interval of 18.5% on the elimination constant and Fasanmade et al. [44] with an effect of treatment phase (induction/maintenance) of 18.3% on clearance. Residual variability was described using proportional [33,35,39,48], additive [38,41,42], or a combined error model [30–32,34,36,37,40,43–47,49].

### Patient characteristics

The study population included a total of 298 patients, after exclusion of concentrations over the upper limit of quantification from the analysis due to their low frequency in the overall dataset (5.15%). Concentrations below the lower limit of quantification were accounted for using the M3 method [50]. Characteristics of all patients and each defined subpopulation are summarized in Тable 2. For a couple of covariates, values at certain timepoints were, however, missing due to retrospective data collection (albumin in 89.9% of timepoints, faecal calprotectin in 63.6%), while for some (weight at baseline, C-reactive protein) most values were present (missing in up to 15% of timepoints). All other covariates included in this research were present at all timepoints (Table 2).

**Table 2:** Characteristics of the patient population used for model evaluations.

Characteristic [units] number (%)/median (Q1-Q3)		ALL (N = 298)	ASUC (N = 45)	CD (N = 116)	FIST (N = 59)	UC (N = 78)
Demographic	**Gender [male]**	166 (55.7)	28 (62.22)	64 (55.17)	38 (64.41)	36 (46.15)
	**Age [year]**	37 (27-45)	39 (28-47)	35 (26-43)	39 (26-48.5)	36.5 (28-44)
	**Body weight [kg]**	75 (64-82)	75 (67-84.5)	77 (63.75-82.25)	75 (64-83)	70 (62.25-78)
Treatment	**IFX dose [mg]**	400 (300-500)	400 (300-500)	400 (300-400)	400 (400-500)	400 (400-500)
	**Time since last dose [days]**	39 (28-56)	37 (28-52)	42 (28-56)	31 (28-49)	42 (28-56)
	**No. of concentrations**	1482	214	540	366	362
	**IFX trough concentration [mg/L]**	6.49 (2.83-11.59)	6.65 (2.81-11.19)	6.642 (2.9-11.5)	6.525 (2.75-11.54)	6.2175 (2.88-11.82)
	**TDM samples during maintenance phase**	1236 (83.4)	177 (82.7)	438 (81.1)	320 (87.4)	301 (83.1)
	**Positive anti-drug antibodies**	57 (3.85)	11 (5.14)	23 (4.26)	9 (2.46)	14 (3.87)
	**Concomitant immunomodulators**	865 (58.37)	123 (57.48)	271 (50.19)	261 (71.31)	210 (69.77)
	**Previous biologic treatment**	73 (24.5)	10 (22.2)	32 (27.6)	16 (27.1)	15 (19.2)
Biochemistry	**C-reactive protein [mg/L]**	1.97 (0.8-4.7)	1.7 (0.82-4)	1.8 (0.7-4.01)	2.8 (1.0-5.85)	1.75 (0.7-4.55)
	**Albumin [g/L]**	42 (37-46)	41 (36.25-45.75)	44 (42-47)	41 (33.5-43)	43.5 (37.5-45.75)
	**Faecal calprotectin [μg/g]**	89 (50-385.5)	99 (50-311)	103 (50-364)	50 (48.65-270.5)	122 (50-477.74)

ALL – overall dataset, ASUC – acute severe ulcerative colitis subpopulation, CD – Crohn’s disease subpopulation, FIST – fistulizing Crohn’s disease subpopulation, Q1 – first quartile; Q3 – third quartile; UC – ulcerative colitis subpopulation.

### Predictive performance of population models

**Fig 3:**
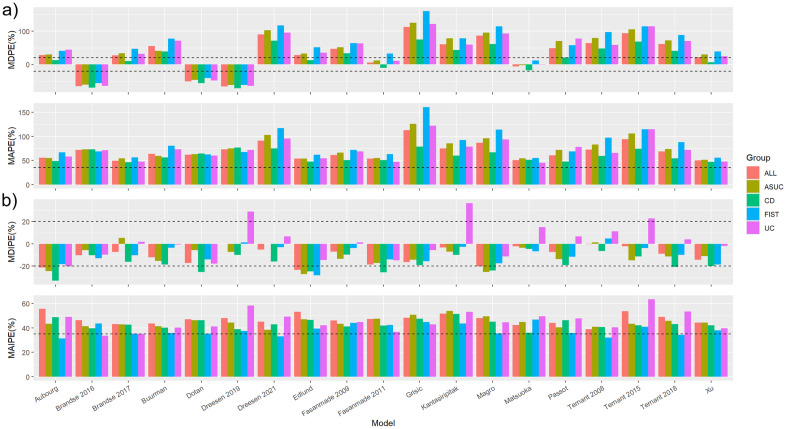
Prediction-based evaluation: a) *a priori* evaluation; b) *a posteriori* Bayesian forecast using pharmacokinetic (PK) infliximab (IFX) models [30, 32–49]. Dashed lines represent the threshold criteria for bias (−20%, 20%) and precision (35%). ALL – overall patients, ASUC – acute severe ulcerative colitis patients, CD – Crohn’s disease patients, FIST – fistulizing Crohn’s disease patients, MAPE – median absolute prediction error accounting for precision, MAIPE – median absolute individual prediction error, MDPE – median prediction error, MDIPE – median individual prediction error, UC – ulcerative colitis patients.

Fig 3 summarizes both *a priori* and *a posteriori* evaluation of nineteen out of the twenty analysed models. The model of Petitcollin et al. [31] was excluded from the graphical representation of the results since the values obtained were much higher compared to the other models which would lead to lower visibility of other results (values for MDPE and MAPE were over a 1000%). These results are consistent with previous evaluation [16].

### A *priori* evaluation

During model evaluation on the overall dataset, the lowest MDPE was achieved by the Fasanmade 2011 (5.59%) and Matsuoka (−6.22%) models [30,44]. None of the models fulfilled the criterion for precision, but the lowest MAPE were observed for Xu (49.8%) and Matsuoka (50.94%) models [30,33]. Therefore, the Matusoka model [30] was identified as the most suitable for the overall dataset (Fig 3a).

*A priori* evaluation of subpopulations gave a clear advantage to Matsuoka model [30] (Fig 3a) both for bias and precision: ASUC – MDPE: −2.69%, MAPE: 54.11%; FIST – MDPE: 12.18%, MAPE: 54.97%; UC – MDPE: −0.58%, MAPE: 45.22%.

In the ASUC subgroup, the Edlund and Xu models demonstrated lower MAPE values, although none fell below 35% threshold for acceptable precision. However, as neither met the bias criterion, Matsuoka model was considered the most suitable overall. The only notable deviation among subpopulations was observed in the CD subgroup, likely due to the frequent inclusion of this patient population in the development of several of the evaluated models. A greater number of models were identified as accurate in terms of covariate-based predictions: Aubourg (12.61%), Brandse 2017 (10.41%), Edlund (13.02%), Fasanmade 2011 (−10.57%), Matsuoka (−18%) and Xu (6.71%) [30,33,37,39,42,44]. The most precise models were Brandse (46.29%) and Xu (47.07%) [33,39].

For *a priori* analysis, the conclusions obtained from %PTAs mainly coincide. Table 3 gives %PTAs for reference value for each dataset and the predicted value for models closest to those values. In FIST the largest deviation from the reference was observed, but the model closest was the one chosen in evaluation [30]. In ASUC, a large deviation from the reference value was observed, and the model with the closest value was the Fasanmade [44], which did not give satisfying results in evaluation. The second closest was the Matsuoka model [30] chosen as the most accurate and precise among all models for this subgroup. In other subgroups, the conclusion is the same as in *a priori* evaluation. Generally, in each subgroup three models [38,40,49] predicted a lower percentage of target attainment compared to the respective reference.

**Table 3:** Probability of target attainment (%PTA) of *a priori* results.

Subgroup	Reference TA (%)	Model	Predicted TA (%)
**ALL**	47.09	Matsuoka [30]	48.31
**ASUC**	31.31	Fasanmade 2011/ Matsuoka [30,44]	37.38/ 37.85
**CD**	48.15	Xu [33]	48.7
**FIST**	32.51	Matsuoka [30]	44.5
**UC**	45.03	Matsuoka [30]	46.69

ALL – overall dataset, ASUC – acute severe ulcerative colitis subpopulation, CD – Crohn’s disease subpopulation, FIST – fistulizing Crohn’s disease subpopulation, TA – target attainment, UC – ulcerative colitis subpopulation.

### A *posteriori* Bayesian forecast

As expected, prediction was improved following the inclusion of informative concentrations (Fig 3b). In the overall dataset, most models met the threshold for acceptable bias, with the lowest MDIPE values of −0.18% for Dreesen 2019 [49] and −0.21% for Ternant 2008 model [48]. However, none of the models fulfilled the predefined criterion for precision. The lowest MAIPE value was 39.41%, observed in Ternant 2008 model [48], identifying it as the most precise among those evaluated, despite not reaching the acceptable threshold.

The most accurate predictive model for ASUC patients was Dreesen 2021 [41], with near zero bias (MDIPE: 0.00099%). As it also had the lowest MAIPE among all evaluated models (38.44%), it was considered the most suitable for this patient subgroup.

For CD patients, the Matsuoka model [30] showed favourable performance after introducing informative concentrations (MDIPE: −4.54%, MAIPE: 36.06%). Interestingly, this was the only subgroup in which this model did not perform optimally in the *a priori* analysis, as other models demonstrated superior predictive performance.

In the FIST subgroup, all models except for Edlund (−28.15%) had MDIPE values within the predefined acceptable range [42]. Three models had MAIPE under 35%: Aubourg (31.46%), Ternant 2008 (32.16%) and Ternant 2018 (34.36%) [34,37,48]. Among them, the Aubourg model was the most precise and had acceptable bias (−18.31%). However, the Ternant 2008 model also demonstrated low bias (MDIPE: 4.89%) and comparable precision, making it a strong candidate for this subgroup as well [37,48].

Lastly, the lowest MDIPE in UC patients was observed for Buurman (−0.53%) and Fasanmade 2009 (1.23%) models [36,43]. However, the only model achieving targets for both bias and precision was the Brandse 2016 model with −9.74% and 33.59%, respectively [38].

**Fig 4:**
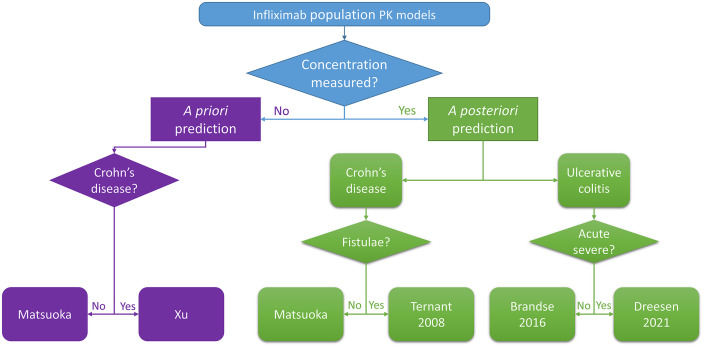
External evaluation results summary. Flowchart and decision tree of selected infliximab (IFX) pharmacokinetic (PK) models [30,33,38,41,48] across different inflammatory bowel disease (IBD) phenotypes and severity.

Based on all evaluation results, Fig 4 presents a flowchart summarizing the favorable performance of IFX PK models for each patient subgroup.

In two cases, similar evaluation parameter values were obtained: during *a priori* evaluation of CD patients and *a posteriori* evaluation of FIST patients. In both cases, two models stood out as adequate based on accuracy and demonstrated comparable precision. Since the MAPE and MAIPE values in both cases differed by less than 1%, the models chosen in the decision tree (Fig 4) were those with lower MDPE/MDIPE, indicating superior accuracy [33,48]. Nevertheless, the alternative models [37,39] should also be viable options considered for these two subgroups.

**Fig 5:**
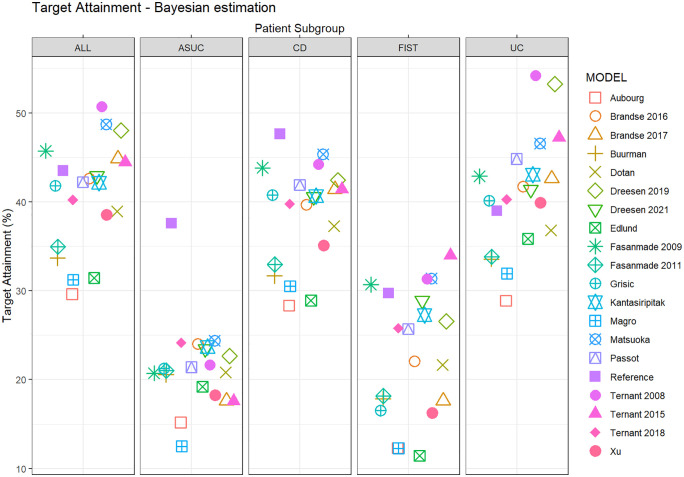
Probability of target attainment (%PTA) analysis from Bayesian estimation.

When we compare results from *a posteriori* analysis with %PTA (Fig 5), some differences were observed. In the CD group, one model [30] showed similar performance across both approaches. In ASUC group, the model performing well in evaluation analysis yielded results closest to the reference [41], although several other models demonstrated comparable %PTA [30,34,38,45,49].

As the evaluation step directly compares predicted and observed concentrations, whereas target attainment analysis reflects only the proportion of concentrations above a predefined threshold, one should rely on the evaluation conclusions (Fig 4), which should nevertheless be interpreted as a general guide rather than a definitive recommendation. However, presented flowchart represents only a guideline based on the real-world dataset.

### Simulation-based diagnostics

**Fig 6:**
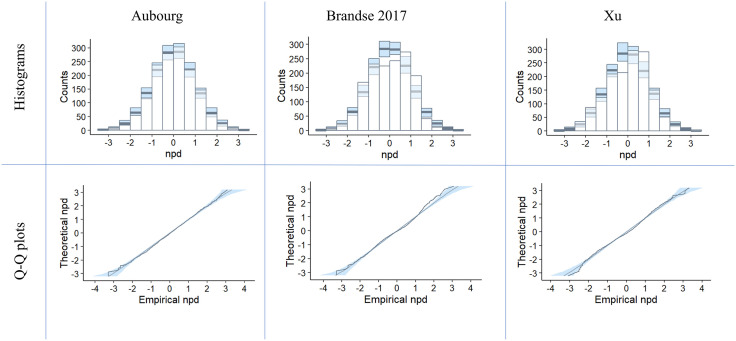
Normalized prediction distribution error (NPDE) charts of the infliximab (IFX) pharmacokinetic (PK) models with satisfactory performance in the overall population [33,37,39].

In the overall dataset NPDE of the Aubourg model [37] was the only one that obeyed a normal distribution with a global test *p*-value > 0.05. Additionally, two models [33,39] had *p*-values over 0.05 for the t-test and Fisher variance test, but did not obey the normal distribution (Fig 6). All other tested models did not achieve set criteria showing significant differences between either the predicted and observed concentrations, variance and did not obey the normal distribution.

After inspection of *p*-values and NPDE charts (S1-S5 Tables and S1-S5 Figs in S1 File) for all four subgroups, it was observed that none of the models performed satisfactorily, with only a few of them that had appropriate *p*-values for Fisher’s variance test, so they are not presented in the article. NPDE is, however, sensitive to dataset characteristics such as sparse and unbalanced sampling, as well as predominantly trough-based observations, which are inherent features of our real-world dataset.

In order to enrich simulation-based analysis, the golden standard to show agreement of data, VPC, have been generated and also placed in the supplementary file (S6-S10 Figs in S1 File). Looking at all 100 graphs, results in certain subgroups are consistent with previous analysis. The VPCs were compared by looking at the deviation of simulation from actual data, the finest model having the least deviations. In the overall dataset, three models [33,39,42] performed better, similar to the NPDE analysis where Brandse and Xu models were also acceptable. Therefore, for simulation purposes, Brandse [39] model was selected. On the subgroup level, conclusions were similar to *a priori* analysis. In CD group, Brandse model was superior, followed by Xu (which was chosen in *a priori* analysis) [33,39]. In both FIST and UC subgroups, Matsuoka model outperformed others, as it did in *a priori* analysis [30]. In the ASUC group, Xu model stood out, followed by Edlund and Matsuoka (which was the model of choice in *a priori* analysis) [30,33,42]. VPC results mainly support the *a priori* analysis conclusions.

## Discussion

We investigated twenty IFX population PK models on a retrospective real-world data from a clinical cohort of 298 patients (Table 2), with the aim of identifying models that may be suitable for MIPD across specific patient subgroups based on both *a priori* and *a posteriori* predictive performance, while also exploring the consistency of different evaluation approaches and metrics.

Based on the results obtained on the overall dataset, the Matsuoka model [30] demonstrated better performance in the *a priori* analysis. This model was developed using real-world data from CD patients, relying exclusively on trough concentrations measured during the maintenance phase. Consequently, a one-compartment model incorporating body weight (BW), ADA and albumin (ALB) adequately fitted the data (Table 1). Similarly, our overall population consists of mainly trough concentrations from the maintenance phase, and the relevant covariates included in the Matsuoka model [30] were also available in our dataset. After inclusion of informative concentrations, model of Ternant 2008 [48] is selected, which is in line with previous report [16]. This two-compartment model was also developed using retrospective monocentric data, and included only the influence of ADA on IFX clearance (Table 1). Simulation-based diagnostics (Fig 5) singled out another model as the only one complying with every statistical test implemented in NPDE analysis. The Aubourg model [37] was also identified in some previous publications as appropriate; however, in one publication [16], it was identified as optimal based on Bayesian diagnostics rather than simulation-based analysis, where Buurman model [36] demonstrated the satisfactory fit. In another study [15], although the Aubourg model [37] showed a good fit, it was ultimately excluded due to differences in the studied population. This divergence between diagnostic methods is not surprising, as the choice of predictive performance metrics should align with the intended purpose of model application. While Bayesian forecasting is adequate when personalized dose adjustment is warranted, NPDE and VPC diagnostic could help select models that are better suited for exploring “what if” scenarios through simulation [22].

Upon reviewing previously published model evaluations, notable discrepancies can be observed both among those studies and in comparison, to the findings of our research. However, our study compared IFX model predictive performance between patient subgroups with various type and severity of IBD. The work by Konecki et al. [15] had a more diverse patient population that included many different indications, so it is to be expected that different models will be identified as appropriate [34,47]; however, as in our NPDE simulation-based analysis, the Aubourg model [37] appeared to show a good fit. Authors Shrapnel and Santacana [13,14] chose the Fasanmade 2011 model [44] on their respective datasets, but our results did not identify this model in either the overall dataset or the subpopulations of interest. The models identified as suitable by Kantasiripitak et al. (*a priori* Edlund [42], *a posteriori*: Aubourg [37], Dreesen 2021 [41], Passot [47], Ternant 2008 [48], simulation-based: Buurman [36]) partially overlap with the models selected in our study population, but are still mostly different. Looking at the results from the VPC simulation-based analysis, different conclusions stem from our study, compared to others mentioned, as well as between them. In line with the conclusion of these authors [16], results suggest that evaluation in different settings should first be performed on their own dataset.

Since *a priori* evaluation relies solely on covariates, and the defined subpopulations were not explicitly included as covariates in the models, it is not surprising that the same model emerged across nearly all subpopulations, mirroring the results observed in the overall dataset [30]. The only population that differed was, surprisingly, the CD patient subgroup maybe due to the fact that some models are developed solely or mostly on this population. The two models that stood out, Xu and Brandse 2017, were both primarily developed using data from CD patients. The more accurate Xu model is suggested in the decision tree (Fig 4), although Brandse 2017 had been slightly, but insignificantly, more precise, neither with the MAPE under 35% [33,39]. Even though Matsuoka model did not outperform, bias was acceptable and precision was similar as for selected models (Fig 3).

Model Dreesen 2021 [41] well suited patients that experienced an episode of ASUC after *a posteriori* analysis, even though it was developed on the CD patient population. Statistically significant covariate included in this model was Crohn’s disease activity index (CDAI), that could indicate that some patients had more severe form of this disease entity, although it is not clear how many. Nevertheless, the fact that the model was developed on moderate to severe CD patients could be the reason why this model was most suitable in ASUC subgroup.

In CD patients, however, Matsuoka model [30], which was optimal for all other subgroups and overall dataset in *a priori* analysis, performed satisfactory after the inclusion of informative concentrations. This model was in fact developed on a CD population of patients in a clinical setting using trough measurements, which was the most similar to our subpopulation of patients.

Patients that had fistulizing disease form had three candidate models as suitable after *a posteriori* evaluation. The most accurate and precise was the Ternant 2008 model [48]. Patient population in this article consisted largely of CD patients, almost half of whom (43.3%) had a high CDAI score consistent with a severe form of the disease that can be similar to the FIST subgroup.

Finally, the model of Brandse 2016 [38] was the most suitable for the UC subgroup, which is consistent with the fact that it was developed using data from UC patients. Additionally, all chosen models incorporated covariates that were available in the evaluation dataset, with the exception of the Dreesen 2021 model [41], which included the CDAI. However, this covariate is not applicable to ASUC patients and would be excluded from analysis anyway. The most frequently included covariates across models were BW, ALB and ADA. These covariates can easily be collected and readily available in clinical setting.

According to the *a posteriori* evaluation, a substantial number of models demonstrated adequate accuracy in each subpopulation, while only a couple of models were accurate enough in the *a priori* analysis. The precision of the models across all subgroups and in both types of analysis was not satisfactory, with only some models reaching the loosened threshold. In each patient subgroup, a different model was selected in *a posteriori* evaluation, which speaks in favour of MIPD combined with TDM depending on the patient disease characteristics. Although these results cannot be generalised, they may provide useful guidance for model selection in clinical settings (Fig 4) and offer a foundation for future studies. The proposed decision tree is, therefore, not a strict recommendation, but a heuristic starting point to support model selection. We acknowledge that this standard methodology does not replace individual patient-specific model selection, and may overlook intra-subgroup variability. Considering recent methodologies, such as model-averaging approaches [16], may enhance predictive performance and robustness in MIPD by accounting for model uncertainty; however, their implementation requires a distinct analytical framework, additional methodological complexity and computational effort, which was beyond the scope of this study that focused on the external evaluation of individual models across clinically defined subgroups as a pragmatic and implementable approach. Future work should compare model averaging with subgroup-based strategies in clinical settings.

A potential limitation of this study is that, although model selection was performed through a structured PubMed search using predefined terms and inclusion criteria, not all published population pharmacokinetic models may have been identified or included. In addition, our analysis incorporated all prior observations within a Bayesian forecasting framework, which may introduce uncertainty, as it assumes stable individual pharmacokinetic parameters over time unless time-varying covariates that capture changes in disease activity are accounted for in the model or not consistently available in dataset. Nevertheless, a violation of assumptions of causality and homogenity in standard population PK models [51] may be a key reason for the emergence of bias in Bayesian forecasting based on multiple measured trough concentrations. Given the retrospective nature of data collection in a clinical setting, there is an inherent risk of errors and a significant proportion of missing data and/or relevant covariates needed for comprehensive analysis. These issues are, however, frequently tackled with in an everyday clinical setting, thus our dataset better describes real-world scenarios. Some covariates that were unavailable in our database (various endoscopic scores, fat-free mass, other indications) or missing in a high percentage of timepoints (albumin level, faecal calprotectin in stool) which may have affected model performance. Models depending on these covariates could appear less accurate when values were unavailable, despite appropriate underlying structure. These covariates might also be missing in other clinics after patients’ check-ups, since they are not routinely obtained, so in model building the choice of covariates should be adapted to the effortlessness of collection and availability of data on patient follow-up. Another limitation is that the study was conducted at a single clinical centre, which may affect the generalizability of the findings. Future research should aim to validate these results across multiple centres and larger patient cohorts to assess reproducibility and confirm whether similar conclusions hold in broader clinical contexts.

## Conclusions

The predictive performance of the evaluated models in the *a priori* analysis was limited, indicating that covariate-based predictions alone may not be sufficient to support individualized IFX dosing in IBD patients. While *a posteriori* approaches naturally improve accuracy by incorporating observed concentrations, our findings support that the choice of PK model, tailored to disease phenotype and severity, plays a significant role in optimizing predictive performance.

This study highlights that no single model performs consistently across all IBD subgroups. Instead, certain models may be better suited for specific clinical contexts, such as CD with or without fistula, or UC with or without prior/current acute severe flares. These insights support a subgroup-driven approach to model selection as a meaningful step toward effective MIPD. In addition, our study shows that no single metric is sufficient to comprehensively evaluate model performance. Hence, a combination of complementary evaluation metrics and approaches is required to adequately assess predictive accuracy, precision, and clinical relevance.

## Supporting information

S1 File S1 Fig. Normalized prediction distribution error (NPDE) histograms of the infliximab (IFX) pharmacokinetic models that performed the best in the overall (ALL) population. S1 Table. Adjusted *p*-values of statistical tests used to test the normality of the normalized prediction distribution error (NPDE) distribution in the overall (ALL) population. S2 Fig. Normalized prediction distribution error (NPDE) histograms of the infliximab (IFX) pharmacokinetic models that performed the best in the acute severe ulcerative colitis (ASUC) subpopulation. S2 Table. Adjusted *p*-values of statistical tests used to test the normality of the normalized prediction distribution error (NPDE) distribution in the acute severe ulcerative colitis (ASUC) subpopulation. S3 Fig. Normalized prediction distribution error (NPDE) histograms of the infliximab (IFX) pharmacokinetic models that performed the best in the Crohn’s disease (CD) subpopulation. S3 Table. Adjusted *p*-values of statistical tests used to test the normality of the normalized prediction distribution error (NPDE) distribution in the Crohn’s disease (CD) subpopulation. S4 Fig. Normalized prediction distribution error (NPDE) histograms of the infliximab (IFX) pharmacokinetic models that performed the best in the fistulizing Crohn’s disease (FIST) subpopulation. S4 Table. Adjusted *p*-values of statistical tests used to test the normality of the normalized prediction distribution error (NPDE) distribution in the fistulizing Crohn’s disease (FIST) subpopulation. S5 Fig. Normalized prediction distribution error (NPDE) histograms of the infliximab (IFX) pharmacokinetic models that performed the best in the ulcerative colitis (UC) subpopulation. S5 Table. Adjusted *p*-values of statistical tests used to test the normality of the normalized prediction distribution error (NPDE) distribution in the ulcerative colitis (UC) subpopulation. S6 Fig. Visual Predictive Check (VPC) of the evaluated models in the overall dataset (ALL); concentrations (µg/mL) vs. time since last dose (days). Solid and dashed lines represent the median, 5th and 95th percentiles of the observed data with shaded 95% confidence intervals of the simulation-based prediction intervals. S7 Fig. Visual Predictive Check (VPC) of the evaluated models in the acute severe ulcerative colitis (ASUC) subpopulation; concentrations (µg/mL) vs. time since last dose (days). Solid and dashed lines represent the median, 5th and 95th percentiles of the observed data with shaded 95% confidence intervals of the simulation-based prediction intervals. S8 Fig. Visual Predictive Check (VPC) of the evaluated models in the Crohn’s disease (CD) subpopulation; concentrations (µg/mL) vs. time since last dose (days). Solid and dashed lines represent the median, 5th and 95th percentiles of the observed data with shaded 95% confidence intervals of the simulation-based prediction intervals. S9 Fig. Visual Predictive Check (VPC) of the evaluated models in the fistulizing Crohn’s disease (FIST) subpopulation; concentrations (µg/mL) vs. time since last dose (days). Solid and dashed lines represent the median, 5th and 95th percentiles of the observed data with shaded 95% confidence intervals of the simulation-based prediction intervals. S10 Fig. Visual Predictive Check (VPC) of the evaluated models in the ulcerative colitis (UC) subpopulation; concentrations (µg/mL) vs. time since last dose (days). Solid and dashed lines represent the median, 5th and 95th percentiles of the observed data with shaded 95% confidence intervals of the simulation-based prediction intervals.
(ZIP)
