# Supplementary material for: Pharmacokinetic model selection for infliximab based on inflammatory bowel disease phenotype and severity: Toward model-informed precision dosing
Source: PLoS One. 2026 Jul 24;21(7):e0352967. doi: 10.1371/journal.pone.0352967 (PMC13399310; doi:10.1371/journal.pone.0352967)
Supplement: S1 File — Normalized prediction distribution error (NPDE) histograms of the infliximab (IFX) pharmacokinetic models that performed the best in the overall (ALL) population. S1 Table. Adjusted p-values of statistical tests used to test the normality of the normalized prediction distribution error (NPDE) distribution in the overall (ALL) population. S2 Fig. Normalized prediction distribution error (NPDE) histograms of the infliximab (IFX) pharmacokinetic models that performed the best in the acute severe ulcerative colitis (ASUC) subpopulation. S2 Table. Adjusted p-values of statistical tests used to test the normality of the normalized prediction distribution error (NPDE) distribution in the acute severe ulcerative colitis (ASUC) subpopulation. S3 Fig. Normalized prediction distribution error (NPDE) histograms of the infliximab (IFX) pharmacokinetic models that performed the best in the Crohn’s disease (CD) subpopulation. S3 Table. Adjusted p-values of statistical tests used to test the normality of the normalized prediction distribution error (NPDE) distribution in the Crohn’s disease (CD) subpopulation. S4 Fig. Normalized prediction distribution error (NPDE) histograms of the infliximab (IFX) pharmacokinetic models that performed the best in the fistulizing Crohn’s disease (FIST) subpopulation. S4 Table. Adjusted p-values of statistical tests used to test the normality of the normalized prediction distribution error (NPDE) distribution in the fistulizing Crohn’s disease (FIST) subpopulation. S5 Fig. Normalized prediction distribution error (NPDE) histograms of the infliximab (IFX) pharmacokinetic models that performed the best in the ulcerative colitis (UC) subpopulation. S5 Table. Adjusted p-values of statistical tests used to test the normality of the normalized prediction distribution error (NPDE) distribution in the ulcerative colitis (UC) subpopulation. S6 Fig. Visual Predictive Check (VPC) of the evaluated models in the overall dataset (ALL); concentrations (µg/mL) vs. [file pone.0352967.s001.zip › S4 Table.docx]

**S4 Table. Adjusted *p*-values of statistical tests used to test the normality of the normalized prediction distribution error (NPDE) distribution in the fistulizing Crohn’s disease (FIST) subpopulation.**

| **Model** | **t-test** | **Fisher’s variance test** | **Shapiro-Wilk test** | **Global test** |
| --- | --- | --- | --- | --- |
| [Aubourg](https://pubmed.ncbi.nlm.nih.gov/26369535/) | 5.92e-98 | 9.71e-11 | 4.93e-33 | 5.92e-98 |
| [Brandse 2016](https://pubmed.ncbi.nlm.nih.gov/26545802/) | 2.53e-186 | 1 | 1.45e-20 | 2.53e-186 |
| [Brandse 2017](https://pubmed.ncbi.nlm.nih.gov/28195852/) | 8.9e-69 | 0.204 | 1.78e-37 | 8.9e-69 |
| [Buurman](https://pubmed.ncbi.nlm.nih.gov/26113313/) | 8.07e-215 | 0.000293 | 6.01e-22 | 8.07e-215 |
| [Dotan](https://pubmed.ncbi.nlm.nih.gov/25358062/) | 7.09e-116 | 1 | 3.26e-35 | 7.09e-116 |
| [Dreesen 2019](https://pubmed.ncbi.nlm.nih.gov/30634202/) | 1.19e-48 | 9.92e-09 | 1.37e-33 | 1.19e-48 |
| [Dreesen 2021](https://pubmed.ncbi.nlm.nih.gov/32415677/) | 3.6e-150 | 0 | 2.75e-35 | 0 |
| [Edlund](https://pubmed.ncbi.nlm.nih.gov/27739011/) | 8.82e-273 | 7.52e-99 | 2.91e-23 | 8.82e-273 |
| [Fasanmade 2009](https://pubmed.ncbi.nlm.nih.gov/19756557/) | 1.11e-198 | 6.13e-34 | 8.11e-13 | 1.11e-198 |
| [Fasanmade 2011](https://pubmed.ncbi.nlm.nih.gov/21741088/) | 1.11e-157 | 5.93e-13 | 7.96e-12 | 1.11e-157 |
| [Grisic](https://pubmed.ncbi.nlm.nih.gov/33184852/) | 2.5e-146 | 1.86e-05 | 2.33e-11 | 2.5e-146 |
| [Kantasiripitak](https://pubmed.ncbi.nlm.nih.gov/33604964/) | Not available | Not available | Not available | Not available |
| [Magro](https://academic.oup.com/ecco-jcc/article/18/7/1102/7579347?login=false) | 7.55e-156 | 0.477 | 3.71e-31 | 7.55e-156 |
| [Matsuoka](https://pubmed.ncbi.nlm.nih.gov/31657711/) | 1.52e-99 | 1 | 5.8e-34 | 1.52e-99 |
| [Passot](https://pubmed.ncbi.nlm.nih.gov/27589009/) | 5.22e-150 | 0.56 | 2.75e-27 | 5.22e-150 |
| [Petitcollin](https://pubmed.ncbi.nlm.nih.gov/30860594/) | 3.21e-41 | 1 | 7.32e-35 | 3.21e-41 |
| [Ternant 2008](https://pubmed.ncbi.nlm.nih.gov/18641542/) | 4.8e-65 | 0 | 1.22e-23 | 0 |
| [Ternant 2015](https://pubmed.ncbi.nlm.nih.gov/25516415/) | 5.25e-134 | 0 | 5.89e-29 | 0 |
| [Ternant 2018](https://pubmed.ncbi.nlm.nih.gov/29236229/) | 2.58e-143 | 1 | 4.14e-26 | 2.58e-143 |
| [Xu](https://accp1.onlinelibrary.wiley.com/doi/10.1177/2160763X12454673) | 8.88e-222 | 1.49e-18 | 1.3e-08 | 8.88e-222 |
